# Dual Fluorescence Splicing Reporter Minigene Identifies an Antisense Oligonucleotide to Skip Exon v8 of the *CD44* Gene

**DOI:** 10.3390/ijms21239136

**Published:** 2020-11-30

**Authors:** Sachiyo Fukushima, Manal Farea, Kazuhiro Maeta, Abdul Qawee Mahyoob Rani, Kazumichi Fujioka, Hisahide Nishio, Masafumi Matsuo

**Affiliations:** 1 Department of Pediatrics, Kobe University Graduate School of Medicine, Kobe 650-0017, Japan; sachi4@med.kobe-u.ac.jp (S.F.); fujiokak@med.kobe-u.ac.jp (K.F.); 2 Research Center for Locomotion Biology, Kobe Gakuin University, Kobe 651-2180, Japan; manalr44@reha.kobegakuin.ac.jp (M.F.); maeta@reha.kobegakuin.ac.jp (K.M.); rani@reha.kobegakuin.ac.jp (A.Q.M.R.); nishio@reha.kobegakuin.ac.jp (H.N.); 3 KNC Department of Nucleic Acid Drug Discovery, Faculty of Rehabilitation, Kobe Gakuin University, Kobe 651-2180, Japan; 4 Department of Occupational Therapy, Faculty of Rehabilitation, Kobe Gakuin University, Kobe 651-2180, Japan

**Keywords:** antisense oligonucleotide, minigene, exon skipping, splicing, dual fluorescence, *CD44*

## Abstract

Splicing reporter minigenes are used in cell-based in vitro splicing studies. Exon skippable antisense oligonucleotide (ASO) has been identified using minigene splicing assays, but these assays include a time- and cost-consuming step of reverse transcription PCR amplification. To make in vitro splicing assay easier, a ready-made minigene (FMv2) amenable to quantitative splicing analysis by fluorescence microscopy was constructed. FMv2 was designed to encode two fluorescence proteins namely, mCherry, a transfection marker and split eGFP, a marker of splicing reaction. The split eGFP was intervened by an artificial intron containing a multicloning site sequence. Expectedly, FMv2 transfected HeLa cells produced not only red mCherry but also green eGFP signals. Transfection of FMv2*CD44*v8, a modified clone of FMv2 carrying an insertion of *CD44* exon v8 in the multicloning site, that was applied to screen exon v8 skippable ASO, produced only red signals. Among seven different ASOs tested against exon v8, ASO#14 produced the highest index of green signal positive cells. Hence, ASO#14 was the most efficient exon v8 skippable ASO. Notably, the well containing ASO#14 was clearly identified among the 96 wells containing randomly added ASOs, enabling high throughput screening. A ready-made FMv2 is expected to contribute to identify exon skippable ASOs.

## 1. Introduction

Splicing is a mechanism to remove intron from pre-mRNA to produce mRNA. For accurate mRNA production, splice site is strictly determined by the conserved splicing consensus sequences located at splice donor and acceptor sites. If a nucleotide change is present in these cis-regulatory sequences, a splicing error is highly expected to occur. Although, skipping of an exon is the most common splicing error caused by nucleotide changes [1], exon skipping occurs naturally and has been identified in nearly 90,000 exons in human genes [2]. One benefit of exon skipping is that a given gene can produce a variety of proteins.

Exon skipping therapy was first proposed by us as a treatment of Duchenne muscular dystrophy (DMD), a fatal progressive muscle wasting disease, in which out-of-frame *DMD* mRNA is transformed into in-frame to resume protein production [3,4]. Subsequently, we demonstrated for the first time that an antisense oligonucleotide (ASO) is able to induce *DMD* exon skipping [5]. Since exon skipping is recognized as one of the most promising therapy for DMD, studies on ASO-mediated exon skipping have been actively conducted worldwide. Recently, two and one exon skippable ASOs were officially approved in USA and Japan, respectively for the treatment of DMD [6,7]. With these approvals of ASO as therapeutics, there is an increasing interest in expanding the application of exon skipping to many other diseases where redundant or dispensable exons may be amenable to exon skippable ASO intervention [8].

There are three main aims of exon skipping therapy: gain of function, loss of function and modulation of function. For the gain of function, exon skipping has been applied to transform out-of-frame mutations into in-frame in the *DMD* gene [4] and to eliminate disastrous mutations of the *DYSF, CEP290*, *COL4A5* and *GNTAB* genes [9,10,11,12]. For the loss of function, skipping of an out-of-frame exon of the *MSTN* gene has been reported [13]. For the functional modulation, *ITGA4* exon 27 which encodes the transmembrane domain of integrin alpha 4 was skipped to produce the soluble form [14]. In this stream, we developed an ASO that induced skipping of exon v8 of the *CD44* gene, thereby, destabilizing the association with cysteine/glutamate transporter [15]. As previously reported, the skipping of *CD44* exon v8 leads to increase in the chemosensitivity of cancer stem cells [16,17].

Exon skippable ASOs or small molecular chemicals have been screened mainly by minigene splicing assays [18,19]. On the other hand, the assay has been commonly used in a cell-based in vitro approach for splicing studies of genomic nucleotide changes [20,21]. In this way, a minigene harboring a genomic segment encompassing the target sequence of interest is constructed and transfected into cultured cells. Thereafter, its splicing product is analyzed by reverse transcription (RT)-PCR amplification. The RT-PCR step is accomplished by multiple experimental procedures, such as RNA extraction, cDNA synthesis, PCR amplification, gel electrophoresis and sequencing, consuming several kits and a lot of time. The construction of a hand-made minigene and the assay by RT-PCR have been major hurdles for the applicability of the minigene assay. To overcome this complicated assay, green fluorescence protein (GFP) expression minigene with an intron sequence sandwiched between split GFP cDNA was invented [22]. However, the analysis was not-quantitative due to the lack of an internal standard. Currently, the demand to use splicing reporter minigene for the estimation of the splicing effect of variants is increasing since the number of variants revealed by next-generation sequencing is rapidly increasing [23]. Therefore, it is an urgent issue to establish an uncomplicated splicing assay system.

Here, we constructed a splicing reporter minigene named FMv2. For accurate quantification of the splicing products in a cell-based system, two fluorescence protein sequences were cloned in the minigene. Furthermore, a multicloning site sequence was inserted in the minigene to allow for straightforward replacement of a variety of gene segments to analyze splicing. One of such minigenes FMv2*CD44*v8, with exon v8 of the *CD44* gene, identified an effective ASO capable of inducing exon v8 skipping. Hence, FMv2*CD44*v8 was shown to be applicable for high throughput screening. The ready-made minigene is highly expected to contribute to the identification of exon skippable ASO as well as the clarification of splicing effects of nucleotide changes.

## 2. Results

### 2.1. Construction of a Dual Fluorescence-Based Splicing Reporter Minigene

A dual fluorescence-based splicing reporter minigene was designed to achieve the following objectives; (1) to eliminate the RT-PCR step, the splicing product was assayed by fluorescence microscopy. For this, the fluorescent eGFP coding sequence was split into 5′ and 3′ parts by the insertion of an artificial intron between them. Completion of the splicing reaction was expected to produce the full-length eGFP, confirmed by the production of green fluorescence signals. (2) To enable quantitative analysis, another fluorescent mCherry protein was constitutively expressed, and the red signal was used as a marker for both transfection and expression efficiency. (3) To increase its applicability for rapid cloning of any target sequence, a multicloning sequence was inserted into the artificial intron. As a result, a 1683 bp long sequence encoding two fluorescence proteins was synthesized and the synthesized fragment was inserted into pcDNA3.1/Hygro (+) plasmid to generate a minigene, FM (Supplementary Figure S1). To examine whether the minigene FM works as a splicing reporter as expected, it was transfected into HeLa cells. Red fluorescence was detected in FM transfected cells. Unexpectedly, green fluorescence was not observed. This suggested that splicing did not occur from FM, leading to no mature eGFP mRNA production. However, the corresponding mRNA of the full-length eGFP was obtained by RT-PCR amplification. Unexpectedly, sequencing of the PCR product revealed a 4-bp deletion at the junction of the 5′ and 3′eGFP region (Supplementary Figure S2). This indicated that cryptic splice site activation produced non-functional eGFP mRNA. It was concluded that FM was not suitable for splicing assay.

### 2.2. Construction of the Second Version of Minigene, FMv2

**Figure 1:**
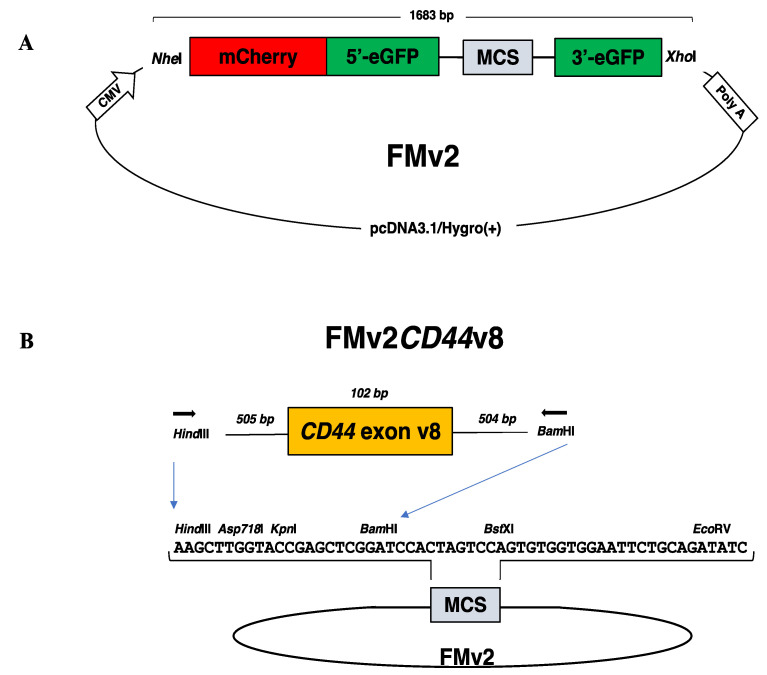
Dual fluorescence-based splicing reporter minigenes. (**A**) The structure of a ready-made dual fluorescence-based splicing reporter minigene is schematically described. An expression vector pcDNA3.1/Hygro(+) encoding the CMV promoter (CMV) and the polyadenylation signal of bovine growth hormone gene (poly A) was inserted with the synthesized 1683 bp long fragment with the use of *Nhe*I and *Xho*I restriction enzymes. The synthesized DNA comprised of sequences of the *Nhe*I restriction enzyme recognition site, mCherry, the 5′ region of eGFP (5′-eGFP), the 5′-end of *DMD* intron 18, the multicloning site (MCS), the 3′-end of *DMD* intron 19, the 3′ region of eGFP (3′-eGFP), and the *Xho*I restriction enzyme recognition site. The MCS sequence is immediately flanked upstream and downstream by the first part of *DMD* intron18 and the last part of *DMD* intron19, respectively. The original construct named as FM was modified to create FMv2 by replacing 15 nucleotides. (**B**) The ready-made minigene (FMv2) was modified to produce FMv2*CD44*v8 as follows. PCR amplified genomic region of exon v8 of the *CD44* gene was inserted into the MCS of FMv2 using *Hind*III and *BamH*I restriction enzymes. The sequence of the MCS is described in the middle together with restriction enzyme recognition sites. Exon v8 of the *CD44* gene (102 bp) and its flanking introns (505 and 504 bps, respectively) were amplified using primers (horizontal arrowhead) with *Hind*III and *BamH*I sequences added at the 5′end.

To eradicate unexpected activation of cryptic splice site in the inserted sequences, all candidate splice sites were abolished from FM by replacing every nucleotide forming GT or AG dinucleotides with other nucleotides, while their coding amino acids were maintained. As a result, 15 nucleotide changes were created in the eGFP sequence to produce FMv2 (Figure 1A).

**Figure 2:**
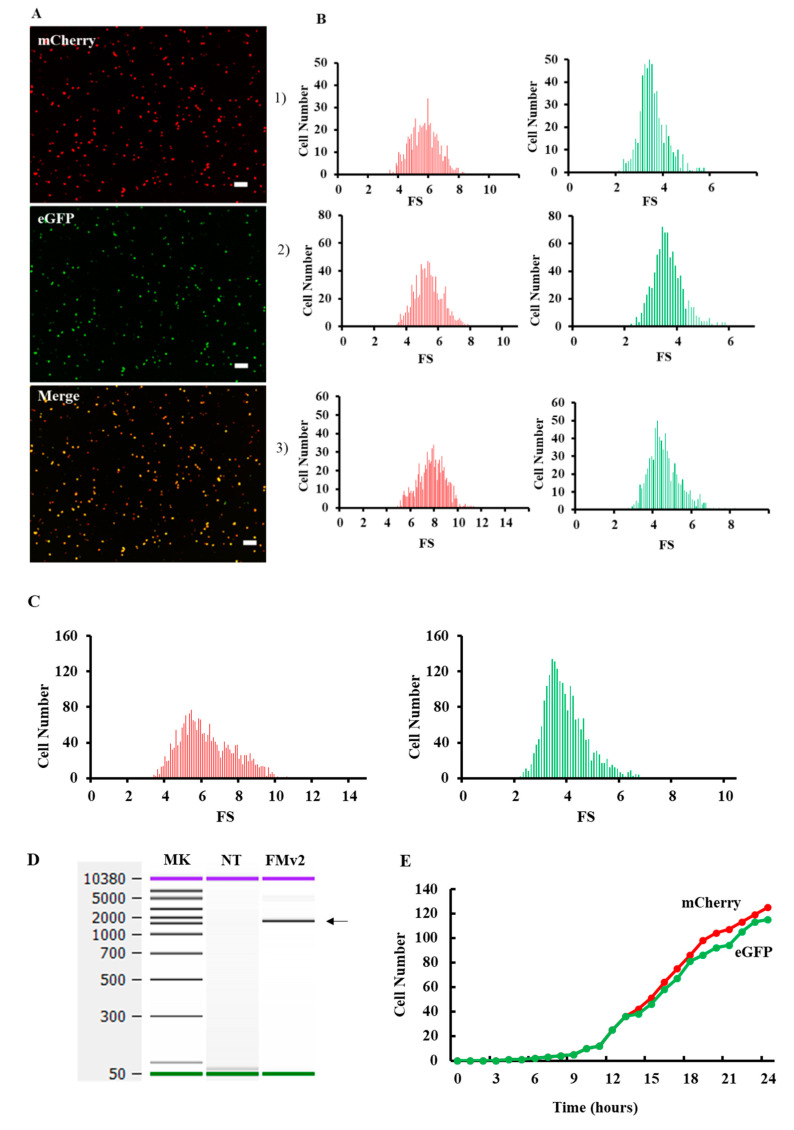
Fluorescence analysis of splicing product in the FMv2 transfected cells. (**A**) Fluorescence images of FMv2 transfected HeLa cells. The FMv2 transfected HeLa cells were scanned for fluorescence by a fluorescence microscope. Representative fluorescence images of HeLa cells are shown. Both red (mCherry) and green (eGFP) signals were clearly detected (top and middle, respectively). The merged figure of red- and green images (merge) produced yellow color (bottom). Scale bars: 100 µm. (**B**) Histograms of fluorescence positive cells. By scanning three wells for each fluorescence, the fluorescence intensity of each cell was obtained. Then, the fluorescence strength (FS) was calculated by the following formula; FS = cell fluorescence intensity (luminance)/cell area (µm^2^). Histograms of FS of red and green fluorescence were constructed in three independent experiments with FS separated into 0.1 FS/bin and are shown. (**C**) Splicing efficiency of FMv2. All histograms of red- and green-positive cells were accumulated, and the accumulated histograms are shown. The number of red- and green-positive cells was 2168 and 2096, respectively. The exon skipping index was calculated to be 96.7. (**D**) RT-PCR analysis of splicing product. Splicing product of FMv2 transfected HeLa cells was analyzed by RT-PCR amplification. The electropherogram of the amplified product is shown. One band was visualized at the position corresponding to 1533 bp (FMv2). Whilst no band was visible in the lane of non-transfected cells (NT). (**E**) FMv2 was transfected into HeLa cells and fluorescence was analyzed at one-hour interval for 24 hrs. The time course of numbers of cell with red-(mCherry) or green-(eGFP) positive signal is shown. Signal-positive cells started to appear at 4 h.

After confirming the sequence of FMv2, it was transfected into HeLa cells for splicing evaluation. Expectedly, not only red but also green signals were microscopically detected, (Figure 2A). Since green signals were produced by eGFP, this indicated production of mature eGFP mRNA by splicing. When the two images were merged, yellow signals were detected, indicating co-expression of red and green signals. To quantify the red- and green-positive cells, histograms representing the fluorescence strength (FS) were constructed (Figure 2B), and the cell number was calculated from the histograms. From three independent experiments, the number of red-positive cells in the wells were 558, 846, and 764, respectively. In contrast, green-positive cells were 549, 830, and 717. The exon skipping index was 98.4, 98.1 and 93.8 and its mean ± SE was 96.8 ± 1.5. As a summary, the number of red- and green-positive cells was 2168 and 2096, respectively (Figure 2C), and the exon skipping index was calculated as 96.7. This indicated that splicing of the FMv2 transcript proceeded almost perfectly.

To verify the completion of splicing at the mRNA level, the minigene transcript in FMv2 transfected cells was analyzed by RT-PCR. The amplification revealed a band that was confirmed as a mature eGFP product by sequencing (Figure 2D). Since no other products were revealed, we deduced that the splicing proceeded perfectly in FMv2 transfected cells. These results matched with the findings disclosed by the fluorescence assay. Hence, it was concluded that FMv2 is a useful minigene for splicing assay. To examine the time-dependency of transcription and splicing reactions, dual fluorescence was analyzed in the FMv2 transfected cells by a time-lapse scanning at one-hour interval for 24 h (Figure 2E). At 4 h, cells expressing red and green signals were detected. We found no clear time lag in the appearance time between the red and green signals. In other words, splicing was completed as transcription occurred. At 24 h the number of green-positive cells was less than that of red-positive cells. Therefore, it was suggested that the exon skipping index was slightly below 100 in this assay system.

### 2.3. Construction of FMv2CD44v8 Carrying Exon V8 of the CD44 Gene

**Figure 3:**
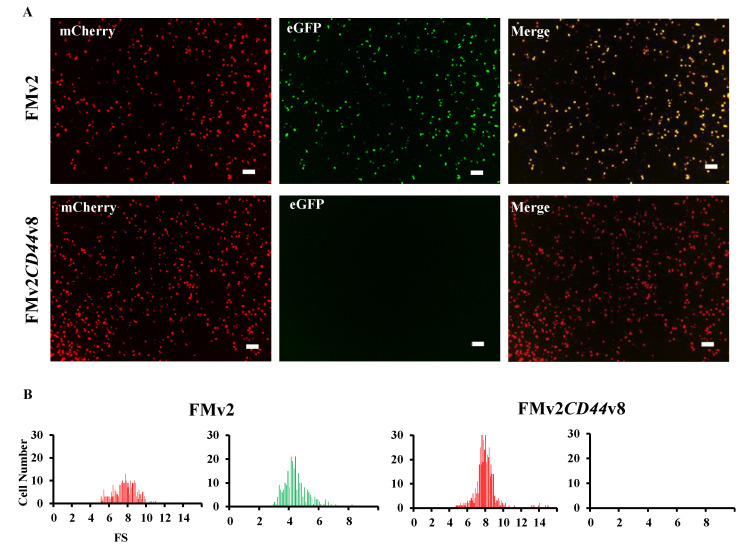
Fluorescence analysis of FMv2*CD44*v8 minigene. (**A**) Fluorescence images of FMv2*CD44*v8 and FMv2 transfected HeLa cells are shown. Red signal was obtained (mCherry) but no green signal (eGFP) was obtained from FMv2*CD44*v8 (bottom), whilst both red and green signals were visualized from FMv2 (top). Yellow signal was detected in the merged image in FMv2 but not in FMv2*CD44*v8 (merge). Scale bars: 100 µm. (**B**) Histograms representing FS are shown. In FMv2*CD44*v8 transfected cells, no cells were green-positive, only red-positive cells were identified (right). In contrast, cells with red- and green-positive signals were identified in FMv2 cells (left).

The ready-made minigene, FMv2, was modified to include the sequence of exon v8 of the *CD44* gene because exon v8 skipping of the *CD44* gene has been reported to be a possible therapy for cancer treatment (16). The genetic sequence of exon v8 and its flanking introns of the *CD44* gene was inserted into the MCS of the FMv2 to construct the FMv2*CD44*v8 minigene (Figure 1B and Supplementary Figure S3). After confirming the nucleotide sequences, FMv2*CD44*v8 was transfected into HeLa cells and fluorescence signals were captured. Expectedly, red-positive cells were identified, but green-positive cells were not (Figure 3A). Contrastingly, in FMv2 transfected cells, both green and red-positive cells were identified. It is suggested that exon v8 incorporation into the mature mRNA disrupted the eGFP coding sequence in FMv2*CD44*v8. Histograms of red and green-positive cells were constructed (Figure 3B). It was observed that the number of red-positive cells was 273 and 473 in FMv2 and FMv2*CD44*v8 transfected cells, respectively. While green-positive cells were 256 in FMv2 transfected cells, and zero in FMv2*CD44*v8 transfected cells. This indicated that exon incorporation occurred in all cells carrying FMv2*CD44*v8. It was concluded that the FMv2*CD44*v8 underwent efficient splicing as designed.

### 2.4. Identification of Exon Skippable ASO Using FMv2CD44v8

Exon skippable ASO was screened using the FMv2*CD44*v8 minigene treated with different kinds of ASOs against the exon v8 sequence. Seven different ASOs were tested (Table 1). Each of them was added to the culture medium in a well of FMv2*CD44*v8 transfected HeLa cells.

**Figure 4:**
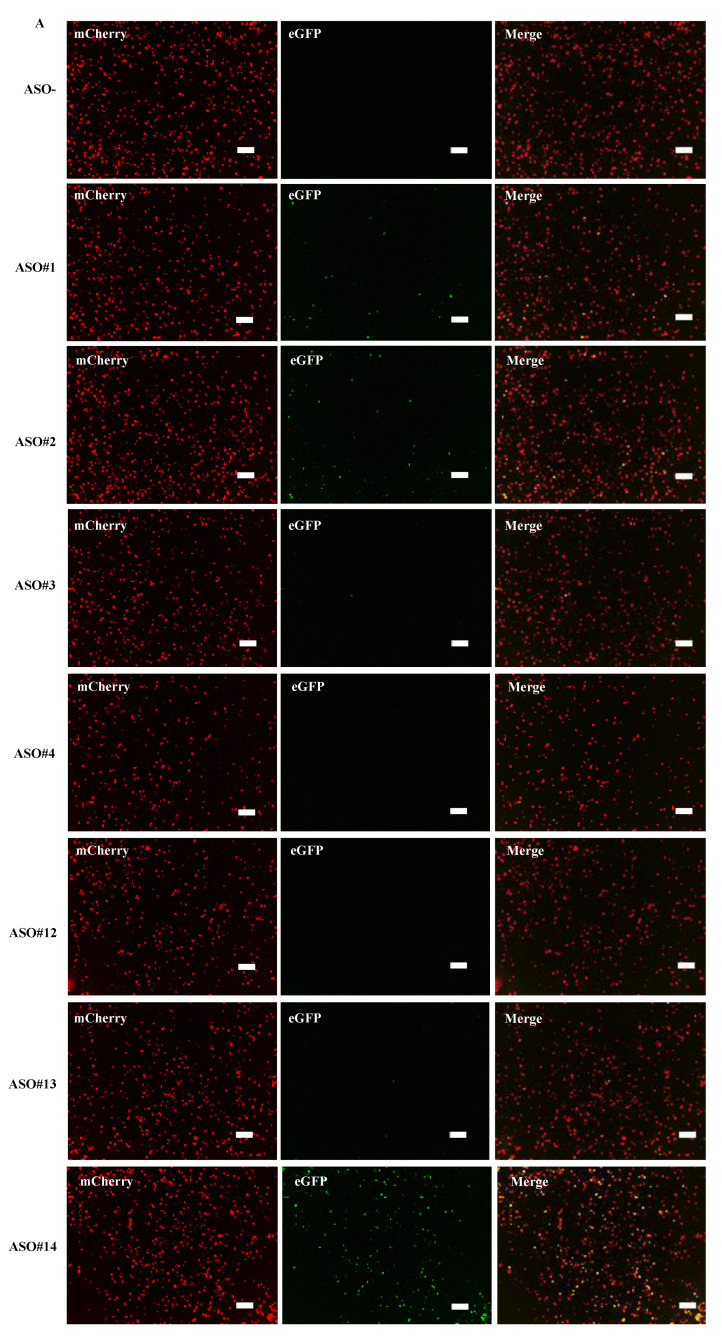

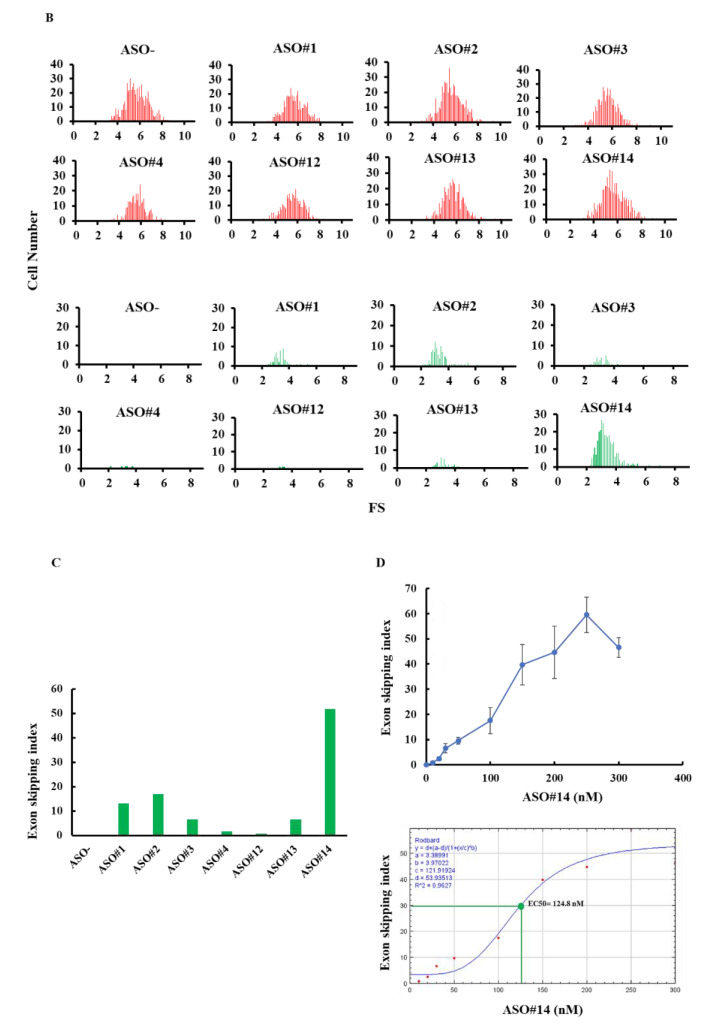
Determination of exon v8 skippable antisense oligonucleotide (ASO) by FMv2*CD44*v8. The reproducibility of our previous results that identified exon v8 skippable ASO by analyzing endogenous mRNA was examined using FMv2*CD44*v8. Seven different ASOs were added to the culture medium of HeLa cells transfected with FMv2*CD44*v8. (**A**) Fluorescence images obtained from transfected cells are shown. Red signal was obtained from every well (mCherry), while green signal was revealed at different levels (eGFP). Yellow signal was detected in limited wells (merge). Scale bars: 100 µm. (**B**) Histograms representing FS are shown. In all, red-positive cells were identified (upper). In contrast, the number of green-positive cells differed from well to well (lower). Non ASO treated cells showed only red signal and not green signal (ASO-). ASO#14 treated cells showed the most abundant green-positive cells (ASO#14). Vertical and horizontal scales indicate cell number and FS, respectively. (**C**) The exon skipping index obtained from the histograms is shown as vertical columns. A high index was obtained in wells containing ASO#14, reaching to 51.7. ASO#14 was shown to be the most efficient to induce exon v8 skipping. (**D**) Dose dependent changes of the exon skipping index with ASO#14 treatment are shown. ASO#14 was added to the culture medium at concentrations ranging from 0 to 300 nM. The index increased linearly with increase in ASO#14 concentrations (top). From this, the half maximal effective concentration (EC_50_) was calculated as 124.8 nM (bottom).

From the results, fluorescence imaging revealed red signals in every well, but the number of green-positive cells differed among wells (Figure 4A). The histograms also revealed a clear difference of green-positive cells between wells (Figure 4B). The exon skipping index was obtained from the histograms (Figure 4C). It demonstrated that, in the well where no ASO was added, the exon skipping index was 0, indicating complete inclusion of exon v8 in the spliced product. In ASO added wells, the exon skipping index ranged from 0 to 51.7 and the highest index was obtained in ASO#14 added wells. It was concluded that ASO#14 is the most potent ASO in inducing exon v8 skipping.

Next, the dose dependency of exon skipping was analyzed using concentrations of ASO#14 ranging from 0 to 300 nM. It was observed that the exon skipping index increased lineally till 250 nM (Figure 4D). From these data, the EC_50_ was estimated as 124.8 nM.

### 2.5. Application of FMv2CD44v8 for a High Throughput Screening

**Figure 5:**
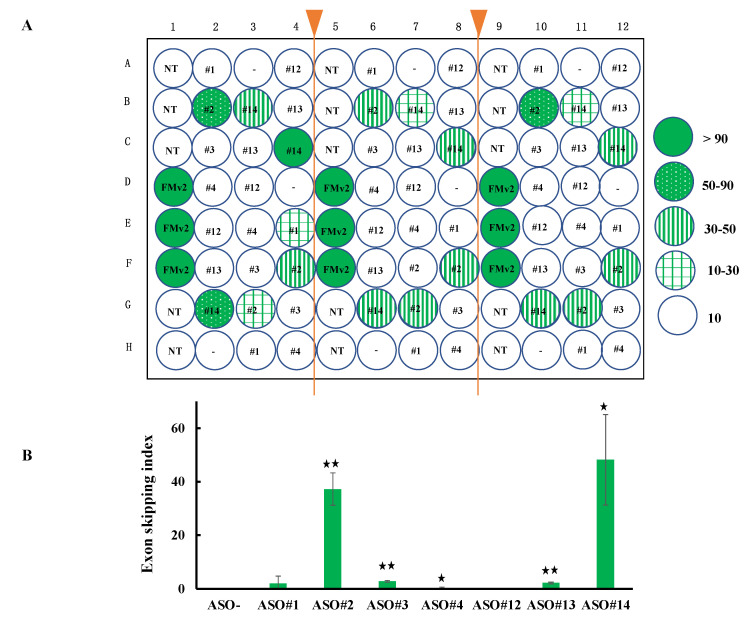
Application of FMv2*CD44*v8 for high throughput screening of exon skippable ASO. (**A**) To examine the applicability of FMv2*CD44*v8 for high throughput screening, a 96 well plate was separated into three parts and seeded with HeLa cells. After transfection of FMv2*CD44*v8, seven different ASOs were randomly and blindly added to the culture medium. Fluorescence was analyzed in each well and the exon skipping index was calculated after the construction of histograms. The index of each well is shown by a color; wells with less than 10, 10–30, 30–50, 50–90, and more than 90 of the index are illustrated by open, plaid green, vertical stripe green, dotted green and dark green circles, respectively. In the center of the circles, the corresponding ASO is indicated. NT denotes non-transfection of minigenes. In three separated regions, the wells with identical treatment disclosed similar index values. This indicated that multiple samples can be analyzed simultaneously. (**B**) The exon skipping index was summarized to reveal ASO-specific activity. The mean values are shown by vertical columns with error bars. Significant differences were detected by comparing the wells with the indicated ASOs and wells without ASO (ASO-) (* *p* < 0.05 and ** *p* < 0.001).

For the applicability of FMv2*CD44*v8 for a high throughput screening, HeLa cells were seeded in 96 wells and transfected with FMv2*CD44*v8. Then, various kinds of ASOs were added into the culture medium of each well. To examine the reproducibility, 96 wells were divided into three blocks (32 wells each) and ASOs were added randomly and blindly in the same pattern in the 32 wells. After 24 hrs, the 96 well plate was scanned. Red signals were observed in every well except for wells without FMv2 or FMv2*CD44*v8. Remarkably, green signals were also recognized in some wells (Figure 5A). After constructing histograms in each well, the exon skipping index was obtained (Figure 5B). The highest index (95-100) was obtained in wells transfected with FMv2. Remarkably enough, the higher index in FMv2*CD44*v8 transfected wells was obtained from wells containing ASO#14 (Figure 5B). The mean ± SE of the exon skipping index of nine wells treated with ASO#14 was 48.2 ± 9.8 and this was significantly higher than that of the wells without ASO addition (*p* < 0.05) (Figure 5B). This multi well analysis successfully identified the most potent ASO. It was concluded that FMv2*CD44*v8 can be used for high throughput screening for exon skipping agents. Altogether, the FMv2 minigene was shown to be easily applicable to different target exons, and effective in the quantitative analysis of splicing.

## 3. Discussion

Dual fluorescence-based splicing reporter minigene named FMv2 was constructed to provide a ready-made minigene for the possible analysis of quantitative splicing. In FMv2, splicing was analyzed microscopically by the capture and quantification of green fluorescence signals of eGFP in a cell based-system using mCherry as a transfection marker. When two images of both red and green fluorescence were merged, yellow signals appeared indicating the co-expression of dual fluorescence. The ready-made FMv2 was easily modified to construct the FMv2*CD44*v8 by inserting a PCR amplified product of exon v8 of the *CD44* gene. This simple way of modification is anticipated to lead to a wider application of other genes. Actually, we have already constructed several modified FMv2 minigenes during a short period of time.

FMv2*CD44*v8 was useful to identify exon skippable ASO. The exon skipping index was easily obtained in each ASO treated well. The highest index was obtained in wells treated with ASO#14 among the wells treated with seven different ASOs. This indicated that ASO#14 has the strongest ability in inducing exon v8 skipping. Notably, this matched with the results obtained from RT-PCR analysis of the endogenous splicing product in AGS gastric cancer cells [24]. It was concluded that minigene splicing analysis using FMv2*CD44*v8 is effective to identify exon skippable ASO. These results indicated that the ready-made FMv2 can be used for drug screening as well as splicing analysis. In the fluorescence assay, splicing analyses were completed within a short time frame and consumed neither kit nor reagent. From these remarkable merits in time and cost, it was concluded that FMv2 is suitable for splicing analysis.

Minigene has been the golden standard to analyze splicing outcome in vitro [20,25]. In the systems, the splicing product is analyzed by RT-PCR amplification of the resultant mRNA. This step requires multiple experimental procedures that need large number of kits, reagents and time and especially, sophisticated techniques. The time- and money-consuming steps hampered the widespread usage of minigene assay. To overcome these disadvantages, a couple of alternative ways were invented. In one, chemiluminescence was applied to monitor splicing products by measuring luciferase activity that indicates completion of splicing [26]. Nevertheless, this procedure still needed a step of assay for luciferase activity. In the others, GFP expression was monitored from the minigene carrying an intron sequence within the GFP cDNA [22,27,28,29]. However, these were not exactly quantitative. Consequently, dual color splicing reporters that encode two fluorescent proteins expressed independently while responding to exon inclusion or skipping have been invented [30,31,32]. In these dual color systems, splicing efficiency was determined by a change of colors. In the latest version, next-generation sequencing system was implemented to replace the PCR amplification step [33]. Thus, a lot of efforts have been put in to improve the minigene assay system for easy amenability and wide-spread usage. We have a long history in producing ASOs that induce skipping of *DMD* exons for the treatment of DMD [5]. During such study, we have produced a ready-made splicing reporter minigene named H492 minigene [34]. This minigene has been utilized not only for analysis of exon skippable agents [11,19] but also for in vitro determination of splicing outcomes caused by nucleotide changes in genetic diseases [35,36,37,38,39,40,41,42]. However, the minigene is facing two drawbacks of RT-PCR amplification and sequencing step. The current FMv2 minigene clearly overcame these drawbacks.

The progress of DNA sequencing technology has revealed a large amount of nucleotide changes of which the assessment of pathological meaning is still pending. Especially, high-throughput DNA sequencing accelerates the pace of discovery of nucleotide changes which are supposed to cause splicing errors. To elucidate the pathogenicity of nucleotide changes, many splicing prediction tools, programs and algorithms have been developed, but none is able to predict splicing effects with a 100% accuracy [20]. Then, splicing error should be disclosed by RNA sample analysis or reasonable alternative minigene assays. In some cases, however, RNA samples from the affected individuals are unavailable, which impedes subsequent transcriptional analysis. As a complementary approach, the demand to use splicing reporter minigene to disclose splicing effects of variants revealed by next-generation sequencing is increasing [23]. FMv2 is highly expected to contribute to the clarification of the pathogenicity of these nucleotide changes.

Exon skipping is recognized as a promising therapy for many diseases [11]. However, it has been a big issue to establish a mass screening system to identify agents to induce skipping of exons. The modified FMv2*CD44*v8 minigene was applied to identify exon skippable ASO using a 96 well plate. In these 96 wells, we found no significant inter-well difference in the numbers of red signal positive cells, indicating a similar efficiency in the transfection. Even though ASOs were randomly and blindly added to the wells, the highest exon skipping index was identified in the wells containing ASO#14. Since fluorescence microscopy has the ability to scan 96 well plates, it is therefore possible to screen 96 different chemicals through a single examination. Such examination would facilitate exon skippable ASO identification.

### Limitation

In this study, a transient expression system was used. To perform high throughput screening, however, it would be better to employ a stably expression system. By so doing, the step of plasmid transfection would be avoided.

## 4. Materials and Methods

### 4.1. Cells

HeLa and AGS cell lines were obtained from ATCC (American Tissue Culture Collection, Manassas, VA, USA) and cultured in Dulbecco’s Modified Eagle’s Medium (08458-16, Nacalai tesque, Kyoto, Japan) supplemented with 10% fetal bovine serum (10270-106, Gibco, thermo fisher scientific, Waltham, MA, USA) and 1% antibiotic-antimycotic (15240-062, Gibco, thermo fisher scientific, Waltham, MA, USA) at 37 °C in a 5% CO_2_ humidified incubator. Cultured cells were rinsed twice with phosphate-buffered saline (11482-15, Nacalai tesque, Kyoto Japan) and then collected using 0.05% trypsin-EDTA (25300-062, Gibco by thermo fisher scientific, Waltham, MA, USA).

### 4.2. Construction of Minigenes

A plasmid, pcDNA3.1/Hygro(+) encoding drug resistant genes as well as CMV promoter and BGH-poly A signal was obtained from Invitrogen (V87020, thermo fisher scientific, Waltham, MA, USA). In this plasmid, the synthesized DNA was inserted after digestion with restriction enzymes, *Nhe*I and *Xho*I (R0131S and R0146S, New England Biolabs, Ipswich, MA, USA). The insert was designed with the following sequences from the 5′ to 3′ ends in this order; *Nhe*I restriction enzyme recognition site, mCherry, 5′ part of eGFP, an artificial intron consisting of the 5′-end of *DMD* intron 18, the multicloning site sequence (MCS), the 3′-end of *DMD* intron 19, 3′ part of eGFP, and *Xho*I restriction enzyme recognition site (Figure 1A). The MCS is immediately flanked upstream and downstream by the first part of *DMD* intron18 and the last part of *DMD* intron19, respectively, as used in H492 minigene [33,43]. The designed 1683 bp long DNA was synthesized by FASMAC Co. Ltd. (Atsugi, Japan). The synthesized DNA was inserted into pre-digested pcDNA3.1/Hygro(+). The construct named FM was sequenced for its integrity and confirmed to consist of the designed sequences. To abolish cryptic splice sites, nucleotide sequences of FM were replaced with other nucleotides at 15 locations to make FMv2 (Supplementary Figure S1).

To evaluate the versatility of the ready-made FMv2 minigene, FMv2 was modified to carry a MCS that can allow a target sequence to be inserted into it. For gene cloning, a region encompassing exon v8 and its flanking introns of the *CD44* gene was PCR amplified from genomic DNA prepared from AGS cells using a set of primers: forward primer CD44v8HindIIIF: 5′-GCGAAGCTTCTAGGTGGTCTTGGATGACG-3′ and reverse primer CD44v8BamHIR: 5′-GCGGGATCCGATCCCGCCACCTTTGTTGA-3′, with underlined *Hind*III and *Bam*HI restriction enzyme site sequences, respectively (Figure 1B). The PCR product was cloned into pre-digested FMv2 with the respective enzymes (*Hind*III-HF: R3104S and *Bam*HI: R0136S, New England Biolabs, Ipswich, MA, USA) to make FMv2*CD44*v8. The construct was confirmed by sequencing.

### 4.3. Minigene Transfection and Expression

Cultured HeLa cells were seeded in a well of 96 well plate at a density of 20,000 cells and 0.1 mL of D-MEM (Nacalai tesque, Kyoto, Japan) was added into each well. After 24 hrs of incubation, 300 ng of FM or FMv2 or 500 ng of FMv2*CD44*v8 minigene solution pre-mixed with 0.4 µL of Lipofectamine 3000 Transfection Reagent (L3000015, Invitrogen, thermo fisher scientific, Waltham, MA, USA) and 0.6 or 1 µL of P3000 (L3000015, Invitrogen, thermo fisher scientific ,Waltham, MA, USA) was added to the culture medium. The media was replaced by FluoroBrite DMEM medium (A1896701, Gibco by Thermo Fisher Scientific, Waltham, MA, USA) at 3 hrs after the transfection. After 24 hrs of incubation, cells were subjected to fluorescence microscopic examination and mRNA analysis.

### 4.4. Fluorescence Analysis

Images were visualized under a fluorescence microscope (BZ-X710, Keyence, Osaka, Japan) with a replaced objective lens (PlanFluor4×, Nikon Instruments Inc., Tokyo, Japan) and a built-in software BZ-analyzer (Keyence, Osaka, Japan). The fluorescence microscope was equipped with a motorized stage and a CCD camera with the dynamic range of 14 bit, and the excitation light emission was kept at the minimal level (low-photobleach mode). The filters included are BZ-X filter GFP (OP-87763, Keyence, Osaka, Japan), and BZ-X filter TexasRed (OP-87765, Keyence, Osaka, Japan). Images were optimized using BZ-Viewer (Keyence, Osaka, Japan). Exposure time of the red (mCherry signal) and green (eGFP signal) was 1 s each. Cell count and quantification of the fluorescence intensity were performed using Hybrid Cell Count (BZ-H3M, Keyence, Osaka, Japan). Fluorescence strength was obtained by the following formula: fluorescence strength (FS) = fluorescence/area of each cell (µm^2^). The exon skipping index was calculated as follows: (the number of green GFP positive cell)/(the number of red mCherry positive cell) × 100. Time-lapse analysis was performed using time-lapse module BZ-H3XT (Keyence, Osaka, Japan) with an incubation chamber (972082, Keyence, Osaka, Japan).

### 4.5. mRNA Analysis

RNA was extracted from harvested HeLa cells seeded in five wells using a high pure RNA isolation kit (Roche Diagnostics, Basel, Switzerland). cDNA was synthesized from 0.5 µg of each total RNA using random primers as described previously [19]. Splicing product was PCR amplified using a set of primers: a T7 promoter forward primer: 5′-TAATACGACTCACTATAGGG-3′ and a BGH reverse primer: 5′-GCTGGCAACTAGAAGGCACAG-3′. PCR amplification was performed in a total volume of 20 µL, containing 2 µL of cDNA, 2 µL of 10× ExTaq buffer (RR001C, Takara Bio, Inc., Shiga, Japan), 0.5 U of ExTaq polymerase (Takara Bio, Inc., Shiga, Japan), 500 nM of each primer, and 200 µM dNTPs (Takara Bio, Inc. , Shiga, Japan). Thirty cycles of amplification were performed on a Mastercycler Gradient PCR machine (Eppendorf, Hamburg, Germany) using the following conditions: 30 cycles of initial denaturation at 94 °C for 3 min, subsequent denaturation at 94 °C for 0.5 min, annealing at 60 °C for 0.5 min, and extension at 72 °C for 1.5 min. Amplified PCR products were electrophoresed using a DNA 7500 LabChip kit (5067-1506 Agilent Technologies, Santa Clara, CA) on an Agilent 2100 Bioanalyzer (Agilent Technologies, Santa Clara, CA, USA) and the separated band was semi-quantified from its density.

For sequencing, PCR-amplified products visualized by agarose gel electrophoresis were excised from the gel with a sharp razor blade, pooled, and purified using a QIAquick gel extraction kit (28706, QIAGEN, Inc., Hilden, Germany). Purified products were sequenced. Sequencing by the Sanger method was performed by FASMAC Co., Ltd. (Atsugi, Japan) using an Applied Biosystems Big Dye Terminator V3.1 (Carlsbad, CA, USA).

### 4.6. Antisense Oligonucleotides

Seven 20 mer chimera oligonucleotides consisting of 2′-O-methyl RNA/2′-O,4′-C-ethylene-bridged nucleic acid (ENA) were analyzed to induce skipping of exon v8 of the *CD44* gene in our previous study (24). To validate our previous results, 7 ASOs were examined for their exon skipping ability using FMv2*CD44*v8. Each ASO synthesized by KNC Laboratories Co., Ltd., Kobe, Japan, was dissolved in water and added to a well at a final concentration of 100 nM.

### 4.7. Determination of EC_50_

The half maximal effective concentration (EC_50_) was calculated from a dose dependent study result using ImageJ software (NIH, Bethesda, MD, USA, http://imagej.nih.gov/ij/).

### 4.8. Statistical Analysis

The Shapiro-Wilk normality test was used to determine if a data set was normally distributed, with a *p* < 0.05 indicating that the data set was non-normally distributed. Statcel 4 software (OMS Publishing Inc., Saitama, Japan) was used for analyses.

## 5. Conclusions

A ready-made minigene (FMv2) amenable to quantitative splicing analysis by fluorescence microscopy was constructed and used for drug screening to reveal an effective ASO. Modification of FMv2 by inserting target exon sequences is expected to contribute to identify exon skippable ASOs.

## Figures and Tables

**Table 1:** Nucleotide sequence of ASOs.

ASO	Sequence
ASO#1	5’-aCTaTgaCTggagTCCaTaT-3’
ASO#2	5’-TTgCagTaggCTgaagCgTT-3’
ASO#3	5’-CaaaCCTgTgTTTggaTTTg-3’
ASO#4	5’-aagaggTCCTgTCCTgTCCa-3’
ASO#12	5’-CCaCCaaaCCTgTgTTTgga-3’
ASO#13	5’-CTgTCCaaaTCTTCCaCCaa-3’
ASO#14	5’-gCgTTgTCaTTgaaagaggT-3’

Upper and lower case letters stand for ENA and. 2’-O-methyl RNA, respectively.

## Supplementary Materials

Supplementary materials can be found at https://www.mdpi.com/1422-0067/21/23/9136/s1.

## Abbreviations

ASO	Antisense oligonucleotide
RT	Reverse transcription
DMD	Duchenne muscular dystrophy
GFP	Green fluorescence protein
ENA	2′-O,4′-C-Ethylene-bridged nucleic acid
EC_50_	Half maximal effective concentration
FS	Fluorescence strength
