# Supplementary material for: Dual Fluorescence Splicing Reporter Minigene Identifies an Antisense Oligonucleotide to Skip Exon v8 of the CD44 Gene
Source: Int J Mol Sci. 2020 Nov 30;21(23):9136. doi: 10.3390/ijms21239136 (PMC7729581; doi:10.3390/ijms21239136)
Supplement: Supplementary file 1 [file ijms-21-09136-s001.pdf]

Supplementary figures and figure legends

```
     NheI ↓ mCherry
FM   GCTAGCGCCACCATGGTGAGCAAGGGCGAGGAGGATAACATGGCCATCATCAAGGAGTTC  60
FMv2 ------------------------------------------------------------

     ATGCGCTTCAAGGTGCACATGGAGGGCTCCGTGAACGGCCACGAGTTCGAGATCGAGGGC 120
     ------------------------------------------------------------

     GAGGGCGAGGGCCGCCCCTACGAGGGCACCCAGACCGCCAAGCTGAAGGTGACCAAGGGT 180
     ------------------------------------------------------------

     GGCCCCCTGCCCTTCGCCTGGGACATCCTGTCCCCTCAGTTCATGTACGGCTCCAAGGCC 240
     ------------------------------------------------------------

     TACGTGAAGCACCCCGCCGACATCCCCGACTACTTGAAGCTGTCCTTCCCCGAGGGCTTC 300
     ------------------------------------------------------------

     AAGTGGGAGCGCGTGATGAACTTCGAGGACGGCGGCGTGGTGACCGTGACCCAGGACTCC 360
     ------------------------------------------------------------

     TCCCTGCAGGACGGCGAGTTCATCTACAAGGTGAAGCTGCGCGGCACCAACTTCCCCTCC 420
     ------------------------------------------------------------

     GACGGCCCCGTAATGCAGAAGAAGACCATGGGCTGGGAGGCCTCCTCCGAGCGGATGTAC 480
     ------------------------------------------------------------

     CCCGAGGACGGCGCCCTGAAGGGCGAGATCAAGCAGAGGCTGAAGCTGAAGGACGGCGGC 540
     ------------------------------------------------------------

     CACTACGACGCTGAGGTCAAGACCACCTACAAGGCCAAGAAGCCCGTGCAGCTGCCCGGC 600
     ------------------------------------------------------------

     GCCTACAACGTCAACATCAAGTTGGACATCACCTCCCACAACGAGGACTACACCATCGTG 660
     ------------------------------------------------------------

     GAACAGTACGAACGCGCCGAGGGCCGCCACTCCACCGGCGGCATGGACGAGCTGTACAAG 720
     ------------------------------------------------------------
     ↓ 5'-eGFP
     GTTAGCAAAGGCGAAGAACTGTTTACCGGAGTTGTCCCCATTCTGGTCGAGCTTGATGGC 780
     -----------T------------------------------------------------

     GACGTGAATGGACACAAGTTTAGCGTCTCAGGAGAAGGAGAAGGAGATGCCACATATGGG 840
     --------------------------AAGT-----------------------G------

     AAACTGACCCTGAAGTTCATCTGCACAACTGGGAAATTGCCTGTGCCTTGGCCGACTCTA 900
     ---------------------------------------------------------T-G

     GTGACAACCCTCACTTATGGCGTGCAGTGTTTCTCCCGATACCCCGATCACATGAAACAG 960
     ------------------------------------A-----------------------

     CATGACTTCTTTAAGTCTGCCATGCCAGAAGGATATGTACAGGAACGTACCATCTTCTTC 1020
     --------------------------------G---------------------------

     AAAGATGACGGCAACTACAAGACGAGGGCTGAAGTGAAGTTTGAGGGCGACACACTCGTC 1080
     --------------------------------G--------------T------------

     AATCGGATTGAGCTGAAAGGCATCGACTTTAAGGAGGATGGGAACATACTTGGTCACAAG 1140
     ------------------------------------------------------------
           ↓ 5'-end of DMD intron 18
     CTGGAATACGTAGGTTATGCATTAATTTTTATATCTGTACTCATTTTGTGCTGCTTGTAA 1200
     -----G------------------------------------------------------
                   ↓ MCS
     ACTCCGTGCTTTGTTAAAGCTTGGTACCGAGCTCGGATCCACTAGTCCAGTGTGGTGGAA 1260
     ------------------------------------------------------------
                 ↓ 3'-end of DMD intron 19
     TTCTGCAGATATCTGGTTTGAAATCATTCATGTGGTGAATACTTTTATAATTAGGATGTG 1320
     ------------------------------------------------------------

     TTGGCTTTCAGATCATTTCTTTCAGTCTGTGGGTTCAGGGGATATATTTAATTATTTTTT 1380
     ------------------------------------------------------------
           ↓ 3'-eGFP
     TCTTTCTAGAACTACAACTCCCATAACGTGTATATCATGGCTGACAAGCAGAAGAATGGC 1440
     ------------------------------------------------------------

     ATAAAAGTGAACTTCAAAATCCGCCATAATATCGAGGATGGTTCAGTCCAACTCGCAGAC 1500
     -----G------------------------------------------------------

     CATTACCAGCAGAACACTCCAATTGGCGATGGACCTGTGTTGCTTCCGGACAATCACTAC 1560
     -----------------------------------------------A------------

     CTGTCAACCCAATCTGCGCTGAGCAAAGACCCCAATGAGAAACGAGATCACATGGTTCTG 1620
     ------------------------------------------------------------
                                                              ↓ XhoI
     CTGGAGTTTGTGACAGCAGCCGGGATTACCCTCGGCATGGACGAACTGTATAAGTAACTC 1680
     ------------------------------------------------------------

     GAG  1683
     ---
```

Supplementary Figure 1　Nucleotide sequences of FM and FMv2 minigenes

Nucleotide sequences of FM and FMv2 minigenes are described. Upper and lower lines indicate FM and FMv2, respectively. Dashes represent identical sequences between FM and FMv2. Arrows indicate borders of segments consisting of *Nhe*I and *Xho*I restriction enzyme recognition sequences, and mCherry, 5'-eGFP, MCS and 3'-eGFP sequences.

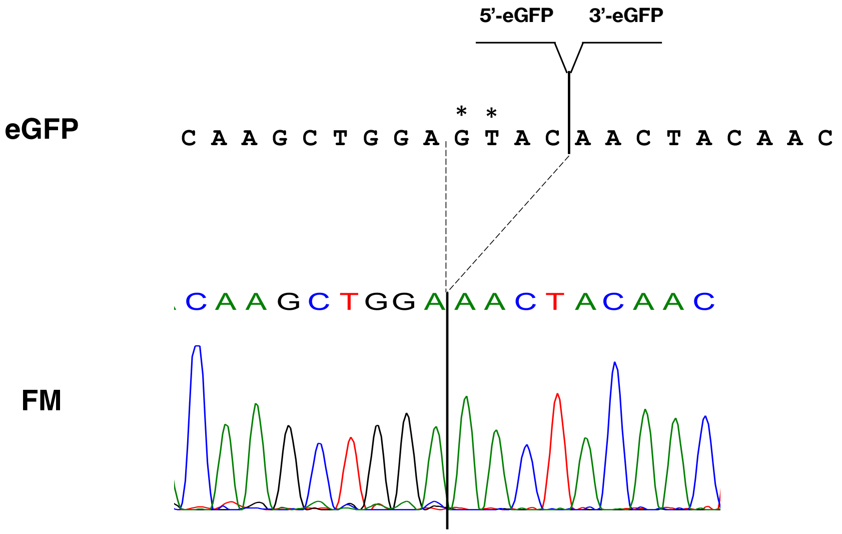

Supplementary Figure 2　Nucleotide sequences at the junction of the 5'-eGFP and the 3'-eGFP

Nucleotide sequences at the junction of the 5'-eGFP and the 3'-eGFP of FM product are shown (FM, lower). The normal eGFP cDNA sequences are described (eGFP, upper). Four nucleotides of GTAC were deleted in the FM product. GT dinucleotides (asterisks) were activated as a splice donor site, producing non-functional minigene transcript. Vertical bars indicate the junction.

AAGCTTCTAGGTGGTCTTGGATGACGATTCTGTTTATTTGCATATTCACTTTGTGAAGAA 60

AAAAAGACTAAAGGGGGGAAGAGGGCTATTTTTAAAAGTCCCTTTGGTTGGTAAGGGGGA 120

GGGGATAAAATGGTGCCTGAGCCAGCACACTTTGTCTTTTCTCCATGTGTCAGATTGCAT 180

GTTTCTATAAAAAAGGCAATGTTTCTGTCTCTCTGCAAGATTTGGGTGTTCCATCATGCA 240

CTCATGTGGAGTCCTTCCAGACTCAGCAAAAACAAACACACAGCATAGCTTTCATATAAC 300

TCGGCCCTTCAAGGAAAGCCAGTTACTTGTTGTGGTGCTCTTTGATGAAACAATAATCTA 360

TCTCAGTTCGGGAGATATAGTCAGTATGTGCTTTCTTTCCTCTTGGCCAGATGTGAATAT 420

TTAAAAAAATCAGCTGTAGACCATAAGCCACCTTCAGGTAGTGGTTTGGGAAATCAAGCA 480

Exon v8

ATAACACTAATATTGATTCCTTCAGATATGGACTCCAGTCATAGTACAACGCTTCAGCCT 540

ACTGCAAATCCAAACACAGGTTTGGTGGAAGATTTGGACAGGACAGGACCTCTTTCAATG 600

ACAACGCGTAAGAATAACGATGCTCAGCCACTTTATTGACTTGTATTCCTGCTTCATCTC 660

TTACTCGCTATTGGCCAAGATGCAGAGCTTTGGTGGTGGAATGGTGCTATGTGGCTTACT 720

TCAGCCCAGAGTGTGAAACTGTCTTCATTGTCTGTATTTCTTGTGGAGGTCTCTCAACCT 780

GATTTTCCCTTCATTTCTTTGTGCCTTCCAGATTTAATGACTTTGTGTTTTAAGAAAAAG 840

TAGGACTCTATTCAACTCCATTTTCATTCTCTAAGTAGTTATGTCAGTGAAACACATGCC 900

CACTTCCAGGATAGTGATTTCTCTATGTTTTAACTGACCATTTCCTTTCAATGTCCATCT 960

TTCCTCCAGCCAGTGCAGAAGAGGGGTAGGATTGCTTGCATGGTTGTAAAAAGACATTTT 1020

TGGGGCCAGATAATCCTAGAGATCACATCAGTTCTCAGAGGAGAGGCAAGGCATGTTATT 1080

GACAATCAACAAAGGTGGCGGGATCGGATCC 1111

Supplementary Figure 3　Nucleotide sequences of *CD44* exon v8 and flanking introns

Nucleotide sequences of the PCR amplified fragment of *CD44* exon v8 and flanking introns

are described. Sequences of *CD44* exon v8 are present between inverted triangles. Arrows indicate primer location and direction.
